# Curcumin-Loaded Mesoporous Silica Nanoparticles Dispersed in Thermo-Responsive Hydrogel as Potential Alzheimer Disease Therapy

**DOI:** 10.3390/pharmaceutics14091976

**Published:** 2022-09-19

**Authors:** Tais de Cassia Ribeiro, Rafael Miguel Sábio, Marcela Tavares Luiz, Lucas Canto de Souza, Bruno Fonseca-Santos, Luis Carlos Cides da Silva, Márcia Carvalho de Abreu Fantini, Cleopatra da Silva Planeta, Marlus Chorilli

**Affiliations:** 1 Department of Drugs and Medicines, School of Pharmaceutical Sciences, São Paulo State University (UNESP), Araraquara 14800-903, Brazil; 2 Department of Biotechnology, Health Sciences Institute, Federal University of Bahia (UFBA), Salvador 40110-902, Brazil; 3 Institute of Physics, University of São Paulo (IF-USP), São Paulo 05508-090, Brazil

**Keywords:** brain disorders, curcuminoids, drug delivery systems, mesoporous silica nanoparticles, neurodegenerative disorder

## Abstract

Alzheimer’s disease (AD) is a neurodegenerative disorder characterized by cognitive and behavioral impairment. Curcumin-loaded mesoporous silica nanoparticles (MSN-CCM) can overcome the drawbacks related to the free curcumin (CCM) clinical application, such as water insolubility and low bioavailability, besides acting over the main causes associated to AD. A thermo-responsive hydrogel is an interesting approach for facilitating the administration of the nanosystem via a nasal route, as well as for overcoming mucociliary clearance mechanisms. In light of this, MSN-CCM were dispersed in the hydrogel and evaluated through in vitro and in vivo assays. The MSNs and MSN-CCM were successfully characterized by physicochemical analysis and a high value of the CCM encapsulation efficiency (EE%, 87.70 ± 0.05) was achieved. The designed thermo-responsive hydrogel (HG) was characterized by rheology, texture profile analysis, and ex vivo mucoadhesion, showing excellent mechanical and mucoadhesive properties. Ex vivo permeation studies of MSN-CCM and HG@MSN-CCM showed high permeation values (12.46 ± 1.08 and 28.40 ± 1.88 μg cm^−2^ of CCM, respectively) in porcine nasal mucosa. In vivo studies performed in a streptozotocin-induced AD model confirmed that HG@MSN-CCM reverted the cognitive deficit in mice, acting as a potential formulation in the treatment of AD.

## 1. Introduction

Alzheimer’s disease (AD) is a progressive neurodegenerative disorder that causes cognitive and behavioral impairment [1]. The deposition of amyloid-Beta plaques, hyperphosphorylation of tau protein (neurofibrillary tangles) in brain regions, and neuronal cell death are the main neuropathological features of AD. In addition, AD is also characterized by neuroinflammation, cholinergic dysfunction, and oxidative damage [2,3,4]. Currently, acetylcholinesterase inhibitors are used to improve the cognitive deficits of AD patients. They act by inhibiting the action of acetylcholinesterase and controlling the levels of acetylcholine available for postsynaptic stimulation [5]. However, the treatment is unable to prevent disease progression [1,6,7]. In addition, the drugs available, such as rivastigmine, donepezil, galantamine, and memantine, present low bioavailability due to the incapacity to cross the blood–brain barrier (BBB), causing side effects, including nausea, vomiting, diarrhea, and hepatotoxicity [8,9,10,11,12]. Therefore, novel therapeutic strategies that prevent or reverse the progression of AD have been required.

Natural products, such as curcumin (CCM), have been studied as promising compounds for the treatment of AD because of their pharmacological properties, including anti-inflammatory, antioxidant, anti-amyloid, and neuroprotective properties [13,14,15,16,17,18]. The antioxidant properties of CCM act on the free radicals present in neurodegenerative processes, protecting the brain from lipid peroxidation and inhibiting hyperphosphorylation of tau protein [19,20]. Moreover, CCM reduces the formation of amyloid-Beta plaques in vivo [20] and exerts action on neuroinflammation by reducing proinflammatory cytokines [21,22]. However, its clinical application is hampered by its water insolubility, extensive metabolization, low bioavailability, and instability in the biological environment [23,24,25].

Considering the limitations mentioned, nanotechnology-based delivery systems can enable the use of natural compounds for the treatment of AD. Specifically, mesoporous silica nanoparticles (MSNs) have shown great potential as drug delivery systems due to their excellent properties, such as physicochemical stability, tunable pore diameter and volume, large surface area, and easy functionalization [26,27,28]. MSNs can encapsulate a high concentration of CCM, increase its solubility and bioavailability in the biological environment, protect it from degradation processes or premature leakage, and favor a controlled drug release [29,30,31,32,33]. Additionally, MSNs exhibit excellent biological properties, including biocompatibility, biodegradability, and low toxicity [26,34,35].

In recent years, the intranasal route has been widely evaluated for drug delivery as a noninvasive route that allows self-administration. In addition, intranasal administration presents advantages, such as a large surface area for drug absorption, highly vascularized epithelium, and enhanced drug bioavailability, owing to the absence of metabolism. Moreover, the intranasal route is an interesting strategy for nose-to-brain drug delivery through the olfactory and trigeminal pathways [12,36]. Despite these advantages, this route presents some limitations, including the short residence time of the formulation in the nasal cavity due to the mucociliary clearance mechanism [37]. For overcoming these drawbacks, the design of thermo-sensitive hydrogels based on poloxamer 407 and chitosan appears as a viable approach to allow intranasal administration of the proposed nanosystem due to their environment-responsive properties (nasal cavity pH and temperature) and enhanced mucoadhesiveness [38,39].

Taking into account the aforementioned and constating that this is an original approach towards neurodegenerative disease treatment, we herein reported the design of a novel formulation containing MSNs as nanocontainers for CCM, dispersed in thermo-responsive hydrogel (HG) for in vitro and in vivo animal AD model evaluation.

## 2. Materials and Methods

### 2.1. Materials

Cetyltrimethylammonium bromide (CTAB, 98%), tetraethyl orthosilicate (TEOS, 99%), l-lysine (98%), styrene, 2,20-azobis (2-methylpropionamide) dihydrochloride (AIBA), ammonium nitrate (NH_4_NO_3_, 98%), chitosan low molecular weight, poloxamer-407, curcumin (77%), and sodium hydroxide solution (NaOH) were purchased from Sigma-Aldrich (St. Louis, MO, USA). Acetonitrile (HPLC grade), acetic acid, and absolute ethanol were purchased from Qhemis. Tween 80^®^ was purchased from Synth (Diadema, SP, BR). Ultrapure water (Millipore, Burlington, MA, USA) was used in all experiments.

### 2.2. Methods

#### 2.2.1. Synthesis of Mesoporous Silica Nanoparticles (MSNs)

The nanoparticles were synthesized according to the methodology described by Nandiyanto et al. 2009 [40]. Briefly, 0.605 g of CTAB was dissolved in ultrapure water (180 mL) at 60 °C under stirring (30 min) in N_2_ atmosphere. After that, the octane (55.8 mL), styrene (105 μL), lysine (0.138 g), TEOS (6.42 mL), and AIBA (0.202 g) were subsequently added to the aqueous solution. The styrene was prewashed with a solution of NaOH (2.5 M). The reaction was kept under stirring at 60 °C in a N_2_ atmosphere for 3 h. Then, the suspension was decanted and purified by centrifugation at a rate of 15,000 rpm for 15 min. The centrifuged nanoparticles were washed with ultrapure water and ethanol. The organic template (CTAB and polystyrene) was removed by refluxing in a mixture of ethanol and 2 g of NH_4_NO_3_ for 1 h at 78 °C [41]. The procedure was repeated twice. The nanoparticle suspension was centrifuged and washed several times with ultrapure water and ethanol and dried under vacuum.

#### 2.2.2. Preparation of CCM-Loaded MSNs (MSN-CCM)

The loading of CCM in the MSNs was carried out according to the methodology adapted from Elbialy et al. (2020) [42]. Briefly, 10 mg of MSNs were added to 1 mL of CCM methanolic solution (2 mg mL^−1^) under stirring (100 rpm) at room temperature and isolated from light for 24 h. Afterwards, the nanosuspension was centrifuged at 5000 rpm for 30 min and washed twice with methanol to remove superficially adsorbed CCM.

Free CCM in the supernatant was quantified by the HPLC method reported by Fonseca et al. (2016) [43]. The encapsulation efficiency (*EE*%) and loading capacity (*LC*%) were calculated according to the following equations:

(1) $$EE\left(\%\right)=\frac{\mathrm{Total}\;\mathrm{amount}\;\mathrm{of}\;\mathrm{curcumin}-\;\mathrm{free}\;\mathrm{curcumin}\;\mathrm{in}\;\mathrm{supernatant}}{\mathrm{Total}\;\mathrm{amount}\;\mathrm{of}\;\mathrm{curcumin}}/100$$

(2) $$LC\left(\%\right)=\frac{\mathrm{Total}\;\mathrm{amount}\;\mathrm{of}\;\mathrm{curcumin}-\;\mathrm{free}\;\mathrm{curcumin}\;\mathrm{in}\;\mathrm{supernatant}}{\mathrm{Total}\;\mathrm{amount}\;\mathrm{of}\;\mathrm{MSNs}\;}/100$$

### 2.3. Physicochemical Characterization of the Nanosystems

#### 2.3.1. Particle Diameter, Polydispersity Index, and Zeta Potential

The hydrodynamic diameter of the nanosystems (MSNs and MSN-CCM) and polydispersity index were determined by dynamic light scattering (DLS) technique. The electrophoretic mobility method was used to measure the zeta potential. The equipment used was the Zetasizer (Malvern Instruments, Worcestershire, UK) at a detection angle of 173°. The nanosystems were diluted 100-fold in ultrapure water and analyzed at 25 °C in triplicate.

#### 2.3.2. Nanoparticle Tracking Analysis (NTA)

The hydrodynamic diameter of the nanosystems was also determined by nanoparticle tracking analysis (NTA). The equipment used was the Nano-Sight NS300 (Malvern Instruments, Worcestershire, UK) equipped with a 532 nm diode laser (green). The nanosystems were diluted 1000-fold in ultrapure water (*n* = 3) and injected with sterile syringes into the sample chamber.

#### 2.3.3. High-Resolution Transmission Electron Microscopy (HR-TEM)

The measurements were performed using the transmission electron microscope JEOL JEM-2100 operating at 200 kV, available at LME IQSC-USP (São Carlos, Brazil). The nanosystems were dispersed in ethanol solution and 3 µL was dropped onto the copper grids. The grids were dried at room temperature and submitted for analysis.

#### 2.3.4. Fourier-Transform Infrared Spectroscopy (FTIR)

The FTIR ALPHA II Spectrometer (Thermo Fisher Scientific, Waltham, MA, USA) was used. The FTIR spectra were obtained in the spectral range from 4000 to 400 cm^−1^, with a resolution of 4 cm^−1^ in the attenuated total reflectance (ATR) mode.

#### 2.3.5. Thermogravimetric Analysis (TGA)

The analysis was carried out using a TG-DSC1 STARe system (Mettler Toledo, Columbus, OH, USA) in a temperature range from 25 to 1000 °C, at a heating rate of 10 °C min^−1^ in air atmosphere dynamic (flow rate = 100 mL min^−1^).

#### 2.3.6. Differential Scanning Calorimeter (DSC)

The analysis was carried out using a TG-DSC1 STARe system (Mettler Toledo, Columbus, OH, USA) in a temperature range from 20 to 250 °C, at a heating rate of 10 °C min^−1^ in air atmosphere dynamic (flow rate = 100 mL min^−1^).

#### 2.3.7. N_2_ Adsorption–Desorption Analysis

Nitrogen adsorption isotherm measurements were performed using Micromeritics ASAP 2020 equipment (Micromeritics Instrument Corporation, Norcross, GA, USA), placed at the Institute of Physics, University of São Paulo. This equipment has two degas stations and one analysis station. The samples were degassed for 24 h. The MSNs samples without CCM were degassed at 200 °C, while the samples with CCM were degassed at 40 °C. The measurements were performed in a liquid nitrogen bath with relative pressures ranging from 10^−6^ to 0.995. The pore surface area was determined by the BET method [44], and the total pore volume and the pore size distribution (PSD) were determined by the BJH-KJS method [45,46] using the adsorption branch. The volume and surface area of micropores were determined by the t-plot method [47].

### 2.4. Preparation of the Thermo-Responsive Hydrogel

The thermo-responsive hydrogel (HG) was obtained according to the cold method described by Schmolka et al. (1972) [48]. First, the chitosan (1% *w*/*v*) was dispersed in a solution of acetic acid (1% *w*/*v*) using a mechanical stirrer (100 rpm) for 3 h. Following complete dispersion, the dispersion was refrigerated (4 °C) and used as a solvent for the dispersion of the poloxamer 407 (18% *w*/*v*). Poloxamer 407 was slowly added to this dispersion and kept in the refrigerator (4 °C) for 24 h. The MSN-CCM was added to the HG at 0.5% (*w*/*v*), remaining under mechanical stirring for 3 h (named as HG@MSN-CCM).

### 2.5. Characterization of the Thermo-Responsive Hydrogel

#### 2.5.1. Rheological Studies

All rheological analyses were carried out in a rheometer model Discovery HR-1 (T.A. Instruments, New Castle, DE, USA) using a cone/plate geometry (40 mm diameter, 2° angle, and gap size of 52 μm).

*(a) Flow Rheometry*

In flow analysis, the shear rate was increased from 0 to 100 s^−1^ and then decreased from 100 to 0 s^−1^ for 120 s each. The analysis was performed at a temperature of 32 ± 0.5 °C. The rheological parameters were calculated by Equation (3) [49]:

(3) $$\tau =K\cdot \gamma^{\eta }$$

where:

*τ* = shear stress (Pa);

*K* = consistency index [(Pa s) n];

*γ* = rate of shear (s^−1^);

η = flow behavior index (dimensionless).

*(b) Oscillatory Rheometry*

First, the viscoelastic region was determined in a range of shear stress from 0 to 10 Pa and a frequency of 1 Hz. Then, frequency sweep analysis was carried out to determine the elastic modulus (G′) and viscous modulus (G″) in the frequency range of 0.1–10.0 Hz at 1 Pa. The analysis was performed at a temperature of 25 ± 0.5 °C and 32 ± 0.5 °C.

*(c) Sol-gel Transition Temperature*

The sol-gel transition temperature (sol-gel) was measured by performing temperature sweeps in the temperature range between 18 and 50 °C at a defined frequency (1.0 Hz) and a rate of heating 3 °C min^−1^.

#### 2.5.2. Texture Profile Analysis

To evaluate the mechanical properties of the hydrogels, a texture analyzer TA-XT2 (MicroSystems) was used with a cylindrical probe (10 mm in diameter). Briefly, the formulations (8 g) were centrifuged to eliminate the air bubbles (at 4000 rpm for 15 min) and placed below an analytical probe. Then, the probe was lowered at a speed of 1 mm s^−1^ until it reached the surface of the hydrogels, up to a predefined depth (10 mm), then returned to the surface at a speed of 5 mm s^−1^. After 5 s, a second compression started in the same parameters. Hardness, compressibility, adhesiveness, and cohesion were obtained from a force–time curve. The analyses were performed in seven replicates at 32 ± 0.5 °C.

#### 2.5.3. Ex Vivo Mucoadhesion

The texture analyzer TA-XT2 (MicroSystems) was used in the adhesion test mode. The nasal mucosa used in the mucoadhesion and permeation assay was obtained from pigs from a local slaughterhouse (Araraquara, São Paulo, Brazil). The mucosa was removed from the nasal cavity with the aid of surgical scissors and scalpel. The nasal mucosa was fixed in the cylindrical probe of the equipment (10 mm diameter). The formulations (8 g) were placed in tubes below the analytical probe and maintained at 32 °C. The probe went down at constant speed (1 mm s^−1^) until the mucosa encountered the formulation’s surface, keeping in contact for 60 s without force under samples. After that time, the nasal mucosa was separated from the hydrogels and detachment force was registered, indicating the bioadhesion strength (N). The analyses were performed in five replicates, at 32 ± 0.5 °C.

#### 2.5.4. Ex Vivo Permeation Studies

The ex vivo permeation assays of CCM from MSN-CCM and HG@MSN-CCM were performed according to a methodology adapted from Rodero et al., (2018) [50], using Microette^®^ Plus (Hanson, Chatsworth, Los Angeles, CA, USA) equipment consisting of glass diffusion cells (7 mL), and porcine nasal mucosa were used. The formulations (MSN-CCM and HG@MSN-CCM) (*n* = 6) were placed (1 mL) in the donor compartment (exposure area of 1.77 cm^2^) and the receptor medium was filled with phosphate buffer (pH 5.4) containing 2% Tween 80 (to maintain the sink conditions) under conditions of temperature (32 ± 0.5 °C) and agitation (300 rpm). Aliquots were collected at predetermined times (0.5, 1, 2, 4, 8, 12, 16, 20, and 24 h) to assess the permeation. The quantification of CCM permeated was measured by HPLC using validated analytical conditions.

### 2.6. In Vitro Cytotoxicity

According to ISO 10993-5 (2009) and USP (2017), qualitative agar diffusion/overlay assay is suitable for liquid, semi-solid, or solid formulations. This assay consists of adding a layer of nutrient-supplemented agar over the monolayer of L929 cells, and the formulation is placed on top of the agar layer. The cells are examined after 24 h of incubation and signs of toxicity are observed after staining with neutral red dye differentiating viable cells from lysed cells [51]. Thus, the study was carried out using L929 cells (fibroblast) cultured in Dulbecco’s modified Eagle’s medium (DMEM) with 10% fetal bovine serum under conditions of controlled temperature (37 ± 0.5 °C) and CO_2_ atmosphere (5%). After reaching 80% of confluence, the trypsin (0.05%, *v*/*v*) was used to detach the cells; then, 8 × 10^5^ cells were seeded into microplates (6-well). To allow adhesion and confluence of 80% of the cells, they were incubated at 37 ± 0.5 °C with 5% CO_2_ for 48 h. After 48 h, the culture medium was removed and each well was washed with phosphate buffer. Then, 1 mL of the culture medium containing agar (0.9%, *w*/*v*) and neutral red dye (0.01%, *w*/*v*) was added to each well. After agar solidification, paper disks were soaked with each formulation and placed in the center of each well. The plates were placed in an incubator at 37 ± 0.5 °C with 5% CO_2_ for 24 h. After incubation, the wells were macroscopically observed, and the formation of a clear halo was measured with a pachymeter. The degree of cytotoxicity was obtained according to ISO 10993-5:2009.

### 2.7. In Vivo Studies

#### 2.7.1. Animals

Seventy-five 30-day-old female Swiss albino mice (weighing approximately 30 g) were used in this study. Animals were supplied from the Central Vivarium of the São Paulo State University—UNESP (Botucatu, São Paulo, Brazil) and were housed in collective plastic cages (five animals per cage) under controlled conditions (the temperature at 24 ± 2 °C and humidity at 50 ± 5%), 12 h light/dark cycle, and free access to water and food. Experimental procedures were performed according to Principles of Laboratory Animal Care published by the US National Institutes of Health (NIH) and were approved by the Institutional Ethical Committee for Use of Animals (protocol CEUA/FCF/CAr number 19/2020).

#### 2.7.2. Intracerebroventricular Injection of Streptozotocin

Stereotaxic surgery was performed for induction of the AD model by intracerebroventricular (i.c.v.) injection of streptozotocin (STZ). First, the animals were submitted to inhalational anesthesia with isoflurane (2%) using a continuous flow anesthesia system. After anesthesia, they were placed in the stereotaxic device and the scalp was anaesthetized with 2% lidocaine hydrochloride and phenylephrine (2 mg kg^−1^) via intradermal administration. Then, the skull was exposed through a unilateral incision and two small bilateral holes were made in ventricles with a low-speed drill at the following co-ordinates from the bregma: 1 mm mediolateral, 0.1 mm anteroposterior, and 3 mm dorsoventral. Finally, i.c.v. injection of STZ (3 mg kg^−1^, the total volume of 5 μL per ventricle^−1^) was performed using a 10 µL Hamilton micro syringe. After the surgical procedure, animals were observed until full recovery.

#### 2.7.3. Experimental Design

**Figure 1:**
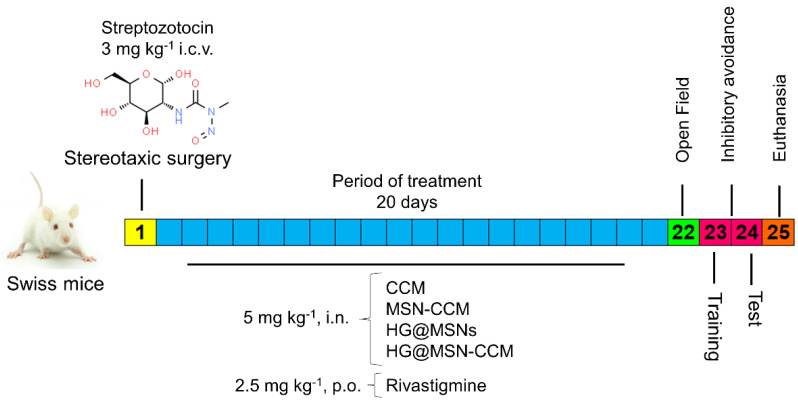
Experimental design. Induction, period of treatment, and behavioral tests performed after treatment and euthanasia.

This study was performed according to the experimental design exhibited in Figure 1. The mice were divided into four groups: (Sham) i.c.v. of saline; (Naive) group healthy and without surgery; positive control i.c.v. of streptozotocin (STZ; 3 mg kg^−1^, 5 μL ventricle^−1^); and negative control i.c.v. of streptozotocin (untreated) (STZ; 3 mg kg^−1^, 5 μL ventricle^−1^). The following treatments were performed: CCM suspension (5 mg kg^−1^, i.n.), MSN-CCM (5 mg kg^−1^, i.n.), HG@MSNs (5 mg kg^−1^, i.n.), HG@MSN-CCM (5 mg kg^−1^, i.n.), and rivastigmine (2.5 mg kg^−1^, p.o.).

#### 2.7.4. Behavioral Assessment

##### Open Field

To assess the possible effect of the treatments performed on locomotor and exploratory behavior, the mice were submitted to the open-field test as described by Capra et al. (2010) [52] with some modifications. Mice were individually placed in an acrylic box (30 × 30 × 30 cm) with the floor divided into 9 equal squares (10 × 10 × 10 cm). Locomotor activity was defined by the number of squares crossed with all 4 paws (crossing), while exploratory behavior was determined by the number of rearing. These parameters were registered for 6 min.

##### Inhibitory Avoidance

The inhibitory avoidance test was performed according to the protocol described by Amoah et al. (2015) [53]. Briefly, the test was carried out in an automated box (25 cm length × 25 cm width × 50 cm height). The floor of this box was constituted by stainless-steel bars separated by 1 cm and connected to an electrical-stimulus generator (intensity of 0.4 mA). On the left side of the box, above the steel bars, was a platform (2.5 cm height × 7.0 cm width × 25 cm length). The mice were placed gently on the platform and, when a mouse stepped down with all four paws on the steel bars, a 3 s 0.4 mA electrical stimulation was applied (training), and the latency to step down placing the four paws on the grid was measured. Then, 24 h after the training, the test session was performed without shock and with step-down latency limited to 180 s.

### 2.8. Statistical Analysis

Results obtained were expressed as mean ± standard deviation (SD). Data were statistically analyzed by the analysis of variance (ANOVA), followed by two-tailed Student’s *t* test. Dunnett’s post hoc test evaluated the differences between the training and testing sections of the in vivo assay.

## 3. Results and Discussion

### 3.1. Physicochemical Characterization of MSNs and MSN-CCM

The particle diameter, zeta potential, and polydispersity index are relevant parameters for biological applications. They represent important characteristics in the stability and biological performance of nanoparticles [54,55,56].

**Figure 2:**
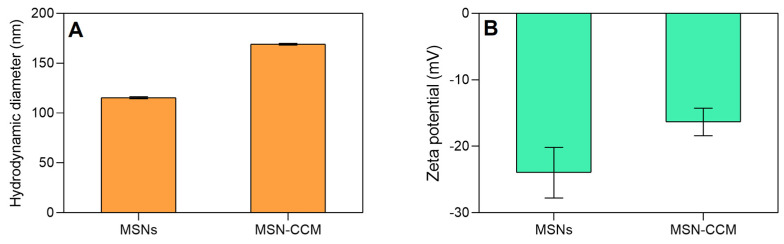
(**A**) Hydrodynamic average diameter and (**B**) zeta potential of the MSNs and MSNs-CCM.

According to the results obtained by DLS (Figure 2A and Figure S1, Supplementary Material), the hydrodynamic diameter of the MSNs (115.63 ± 0.90 nm) was smaller than the MSN-CCM (158.10 ± 9.64 nm) (*p* < 0.05). The hydrodynamic diameter was also analyzed by NTA and values of 116.4 ± 2.5 nm and 167.8 ± 6.7 nm were observed for MSNs and MSN-CCM, respectively (*p* < 0.05) (see histogram in Figure S2). In addition, the 3D graph obtained by NTA shows the intensity of nanoparticles versus hydrodynamic diameter (Figure S3). These data are in agreement and suggest that the loading of CCM increased the hydrodynamic diameter of the nanosystem [57,58].

The polydispersity index represents the particle size distribution. Values below 0.5 indicate a homogeneous size distribution and values greater than 0.5 indicate a heterogeneous size distribution [59]. The polydispersity index increased from 0.26 ± 0.01 to 0.48 ± 0.01 after loading of CCM, indicating a moderate polydisperse profile of CCM-loaded MSNs.

Zeta potential is a characteristic related to the stability of the nanosystem. Values above ±30 mV are considered ideal to prevent the aggregation of nanoparticles [60]. Zeta potential of MSNs decreased from −20.70 ± 1.08 to −16.73 ± 1.91 mV (*p* < 0.05) after loading of CCM (Figure 2B and Figure S4), being considered relatively electrostatically stable nanosystems. Other studies also observed an increase in the hydrodynamic diameter and polydispersity index, as well as a decrease in the zeta potential after loading of CCM [57,58].

**Figure 3:**
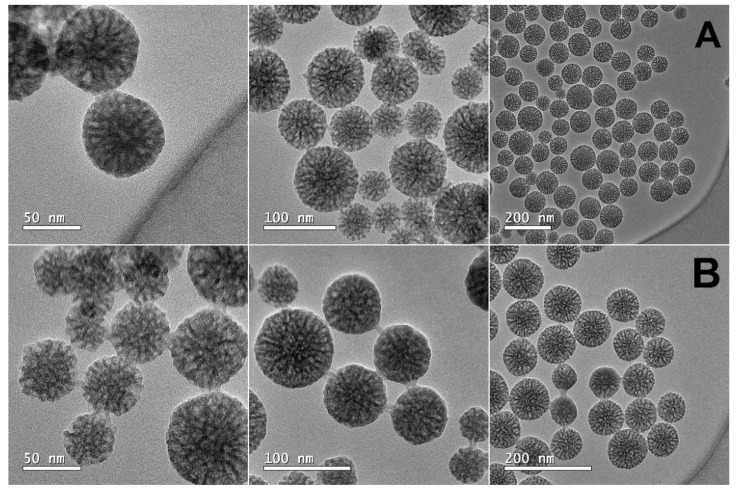
HR-TEM images of (**A**) MSNs and (**B**) MSN-CCM.

HR-TEM was performed to confirm the particle size of MSNs, as well as to assess their mesoporous structure. Figure 3 displays HR-TEM images of the MSNs and MSN-CCM (Figure 3A,B, respectively). Spherical nanoparticles with a random distribution of mesopores in the nanometric range were observed [61,62].

According to microscopy, the nanosystems showed particle average diameter of approximately 60 nm [61,62], corroborating the results found in DLS and NTA. By DLS and NTA analysis, the hydrodynamic diameter was higher due to the hydration layer in aqueous suspension [56]. No changes in shape, particle diameter, and mesoporous structure were observed after loading of CCM.

FTIR analysis was performed to identify characteristic groups of the silica matrix. MSNs present characteristic peaks at 1066 cm^−1^ assigned to silanol groups (Si–OH), and at 805 and 965 cm^−1^ assigned to siloxane groups (Si–O–Si) [40,58,61,62] (Figure S5).

The characteristic groups of CCM are exhibited in Figure S6A. In the FTIR spectrum of MSN-CCM, the band at 3550 cm^−1^ became wider, suggesting the hydrogen bonding between the Si–OH groups and OH groups of CCM [58,63]. In addition, the MSN-CCM presented new peaks at 1526 and 1628 cm^−1^ attributed to C=C groups of CCM. Finally, the interaction between the silica matrix groups and CCM groups can be observed by the shift in the peak position (from 1066 to 1096 cm^−1^) attributed to hydrogen bonds (see Figure S6B) [57,58]. The EE% and LC% were measured to be 87.70 ± 0.05 and 17.54 ± 0.01, respectively, calculated according to Equations 1 and 2.

In other studies, CCM was successfully encapsulated into MSNs showing EE values from 80 to 90% (concentrations from 1.0 to 2.5 mg mL^−1^) [41,51]. MSNs are well known for their large surface area, which allows the encapsulation of high concentration of drugs [25].

**Figure 4:**
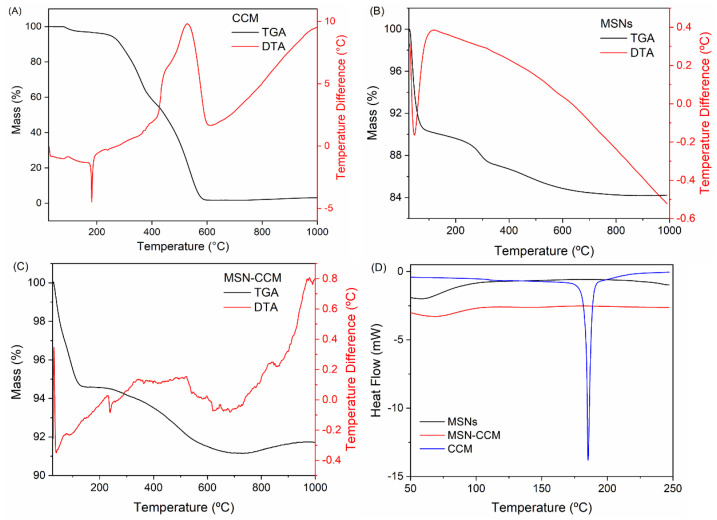
TGA/DTA curves of the (**A**) CCM, (**B**) NSMs, and (**C**) MSN-CCM; (**D**) DSC curves of the CCM, MSNs, and MSN-CCM (blue, black, and red lines, respectively).

Figure 4A exhibits the TGA-DTA curves for CCM with two main events: an endothermic peak at 184 °C assigned to the melting of the CCM and an exothermic event from 300 to 600 °C ascribed to the oxidative degradation [31,64,65]. The TGA–DTA curves of the MSNs are presented in Figure 4B. The TGA-DTA curves of MSNs show two events of weight loss. The first one attributed to the water loss from room temperature to 100 °C and the last one from 200 to 650 °C correspond to the dehydroxylation of the matrix [61,62]. TGA-DTA curves of the MSN-CCM (Figure 4C) display an exothermic event from 250 to 650 °C, which corresponds to the decomposition of CCM present in MSNs matrix. The DSC curve of CCM (Figure 4D, blue line) confirms TGA-DTA results showing an endothermic peak around 184 °C owing to the melting process. However, the DSC curve from MSN-CCM ((Figure 4D, red line) did not show melting peaks characteristic of CCM. Probably, the CCM shifted from a crystalline state to an amorphous state when confined to the mesoporous structure. These results were observed in other studies [42,63,64].

N_2_ adsorption–desorption analysis was performed for MSNs and MSN-CCM (Figure S7). The results (Figure S7A,B) exhibit type IV isotherms with a typical H1 hysteresis loop according to IUPAC classification, classified as mesoporous materials [62,64]. MSNs presented a high surface area, with a large volume of pores and an average pore diameter of 7.2 nm (Table 1). The decrease in surface area (from 745 to 556 m^2^ g^−1^), average pore diameter (from 7.2 to 6.4 nm), and pore volume (from 1.46 to 1.29 nm) corroborate TGA-DTA, DSC, and EE% results, suggesting the successful preparation of CCM-loaded MSNs.

### 3.2. Characterization of Hydrogels

#### 3.2.1. Rheological Studies

**Figure 5:**
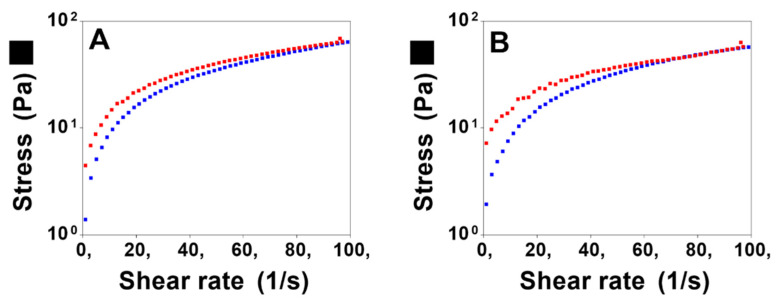
Flow rheograms: HG (**A**) and HG@MSN-CCM (**B**). Red symbol represents the upward curve and blue symbol represents the down curve. Analysis performed at 32 °C.

Hydrogels of poloxamer 407 (18%) and chitosan (1%) containing the MSN-CCM (0.5%) were successfully obtained. The flow properties are shown in Figure 5. Both formulations (HG and HG@MSN-CCM) presented a decrease in viscosity with increasing shear rate (i.e., shear thinning) displaying non-Newtonian flow behavior (*n* < 1) (Table 2) of the pseudoplastic type.

Moreover, they presented thixotropic behavior, since the downward curve returned below the upward curve. It is possible to observe an area of hysteresis indicating time-dependent thixotropy. This indicates that, when the shear stress is removed, the system has the ability to recover their original structure. No significant differences in flow behavior and consistency index were observed after the addition of the nanoparticles to the hydrogel (Table 2).

The oscillatory analysis evaluated the viscous and elastic behavior, providing information on the structural characteristics of the formulations. Thus, the storage modulus represents the energy stored during deformation, a solid-like characteristic of a viscoelastic material. The loss modulus represents the lost energy that cannot be stored, a liquid-like characteristic of a viscoelastic material [65].

**Figure 6:**
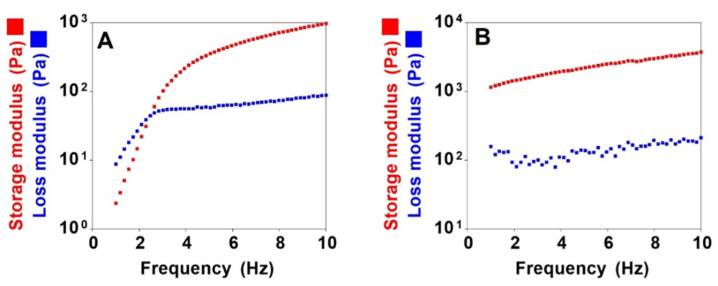
Oscillatory analysis of the HG@MSN-CCM: analysis performed at 25 °C (**A**) and analysis performed at 32 °C (**B**). Storage modulus G′ (red symbols) and loss modulus G″ (blue symbols).

The rheological properties of the HG@MSN-CCM are shown in Figure 6. The results demonstrate the viscoelastic characteristics of the hydrogels at 25 °C where the storage modulus exceeds the loss modulus and an oscillatory frequency dependence is observed (Figure 6A). Nonetheless, at 32 °C, the storage modulus was higher, independent of the oscillatory frequency applied, indicating the formation of a well-structured gel network (Figure 6B).

The gelation process occurs when the storage modulus exceeds the loss modulus (crossover between modulus). To evaluate the phase transition, the loss tangent (Tan d) was used, which is the relationship between the storage modulus and the loss modulus. The abrupt change in Tan d (<1) indicates a greater storage modulus compared with the loss modulus, being indicative of gel formation [66].

**Figure 7:**
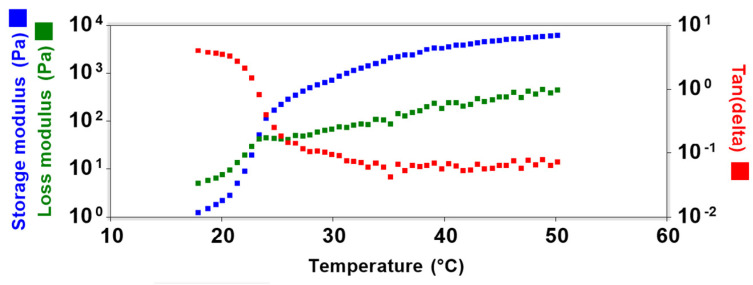
Temperature sweep demonstrating storage modulus G′ (blue symbol), loss modulus G″ (green symbol), and loss tangent (Tan d) (red symbol) of HG@MSN-CCM.

The graph displayed in Figure 7 demonstrates the relationship between the oscillatory modules and Tan D in the temperature range of 18 to 50 °C. Initially, at 18 °C the loss modulus indicates the liquid phase, and, with the increasing temperature, a crossover between the modules was observed, and Tan d < 1 indicates that the phase transition occurred at 23 °C. The storage modulus continues to increase until reaching a stable gel plateau above 32 °C.

#### 3.2.2. Texture Profile Analysis

The mechanical properties of the semi-solid formulations are important to determine the desired application. The ability of the formulation to adhere to a specific surface, for example, the nasal mucosa, is related to the adhesiveness parameter. Meanwhile, the ease of application of the formulation in the nasal region is determined by the hardness parameter. Still, the hardness is related to maximum resistance to deformation. Another important parameter is the compressibility, which is characterized by the work required to compress the formulation, and indicates the spreading of the formulation in the desired location. Finally, cohesion represents the structural recovery of the formulation after its application, which is related to its stability [50,67,68].

The mechanical properties obtained by TPA are shown in Table 3. The formulations did not present hardness and compressibility values at 25 °C, probably due to their liquid state. This result is interesting because the values of hardness and compressibility values should be low to facilitate the administration and spreadability of the hydrogel at the application site [69]. The highest values of hardness, compressibility, cohesion, and adhesiveness were observed at 32 °C, evidencing the excellent mechanical properties of the hydrogels. This result is related to the formation of a well-structured gel network, corroborating the results obtained by rheology. Moreover, the addition of the MSN-CCM did not affect the mechanical properties, since no significant differences were observed.

#### 3.2.3. Ex Vivo Mucoadhesion

Mucoadhesion is related to the interaction between the formulation and the mucin layer. Specifically, the mucoadhesive process occurs due to the intimate contact stage, spreading, and swelling of the formulation in the mucosa. Then, physical or chemical bonds occur between the molecules of the formulation and the mucin, known as the consolidation stage [70,71]. Thus, mucoadhesive formulations, such as hydrogels, are desirable for nasal administration because they are able to overcome the mucociliary clearance mechanism, increasing the formulation residence time in the nasal mucosa and, consequently, the concentration of the drug in situ [72,73,74].

The mucoadhesive property of hydrogels, determined according to the parameters of mucoadhesion strength (S_MA_), corresponds to the maximum detachment force and mucoadhesion work (w_MA_), which corresponds to the total energy involved in the detachment process, are shown in Table 3.

It is known that chitosan is widely studied due to its mucoadhesive properties. Its positively charged amino groups interact with negatively charged sialic acid residues in the mucin layer. On the other hand, poloxamer 407 mucoadhesion is related to covalent bonds with mucin [75].

Antonino et al. (2019) [76] developed a poloxamer-based hydrogel (15–17%) for incorporation of budesonide, an anti-inflammatory used in the treatment of inflammatory diseases. In vitro and ex vivo mucoadhesive studies showed that mucoadhesive strength values increased significantly with the increase in poloxamer concentration, evidencing the excellent mucoadhesive properties of this copolymer.

### 3.3. In Vitro Cytotoxicity

In vitro cytotoxicity assay has been frequently used to assess the biocompatibility of materials for medical use according to the guidelines established by International Standard Organization (ISO 10993-5) [51,77]. This assay is essential, since the cytotoxicity can reduce the safety and therapeutic efficacy of the developed material. The in vitro biocompatibility and cytotoxicity of the formulations were determined by the agar overlay method. This method allows a qualitative analysis of cytotoxicity by indirect contact; thus, it is possible to observe the formation of a clear halo, which indicates dead cells around the sample [50,78].

The results are shown in Table 4. For the negative control, there was no toxicity halo, while, for the positive control (Triton-X), severe cytotoxicity was observed. These results indicate the adequate performance of the assay [50]. It was observed that the MSNs, CCM, and MSM-CCM did not present cytotoxicity. However, the hydrogel formulations (HG, HG@MSNs, and HG@MSN-CCM) showed slight cytotoxicity, with degree of cytotoxicity 1 according to ISO 10993-5:2009.

Regarding biocompatibility, chitosan is a natural polymer considered biocompatible and nontoxic; in addition, it can induce the proliferation of fibroblasts [79,80]. Concerning poloxamer, this copolymer has been extensively studied due to its low toxicity. Moreover, their use as a pharmaceutical excipient is approved by FDA and European Pharmacopoeias [81]. In vitro cytotoxicity studies using human nasal epithelial cells (RPMI 2650) demonstrated the biosafety of poloxamer and chitosan, since no decrease in cell viability was observed [66,73].

In conclusion, the formulations were shown to be biocompatible for L929 cells line at a concentration of 50 μg mL^−1^. Thus, the formulations developed in this work were considered as safe for nasal administration.

### 3.4. Ex Vivo Permeation Studies

**Figure 8:**
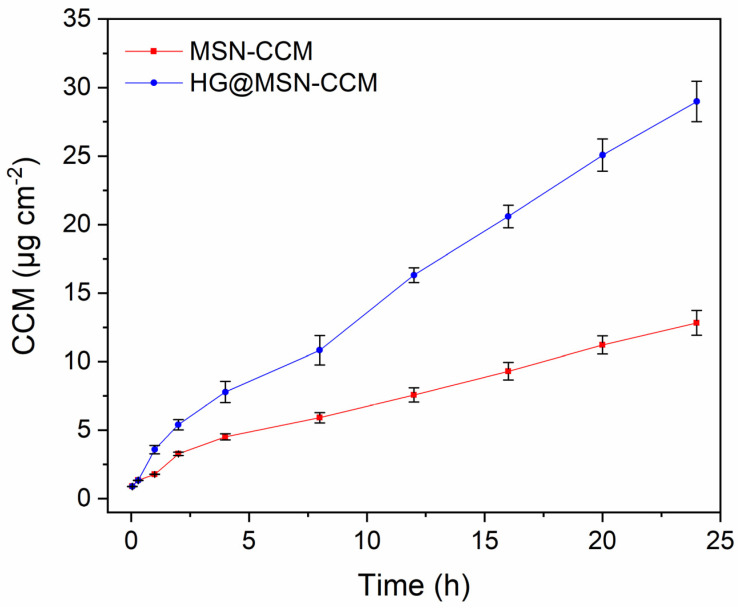
Ex vivo permeation profiles of the CCM from MSN-CCM and HG@MSN-CCM. Profiles are presented as mean ± SD; *n* = 5.

Ex vivo permeation was evaluated over 24 h using porcine nasal mucosa in Franz diffusion cells. CCM suspension was not able to permeate the nasal mucosa. However, MSN-CCM and HG@MSN-CCM formulations (Figure 8) showed permeation of 12.46 ± 1.08 and 28.40 ± 1.88 μg cm^−2^ of CCM, respectively. The higher permeation found from HG@MSN-CCM may be due to penetration-enhancing effects of chitosan, which must have contributed to opening tight epithelial junctions of nasal mucosa, allowing greater permeation of CCM through the mucosa.

Sodd et al. (2014) [82] performed the optimization of a CCM nanoemulsion for intranasal administration. Ex vivo diffusion assays were performed using sheep nasal mucosa. The results showed that the highest permeation was observed for the mucoadhesive nanoemulsion (445.1 ± 37.48 μg cm^−2^), compared to drug solution (302.8 ± 29.86 μg cm^−2^) or CCM nanoemulsion (359.9 ± 36.85 μg cm^−2^). This result was assigned to the mucoadhesive and absorption-enhancing property of chitosan, which is already well-established [37,83,84].

### 3.5. In Vivo

**Figure 9:**
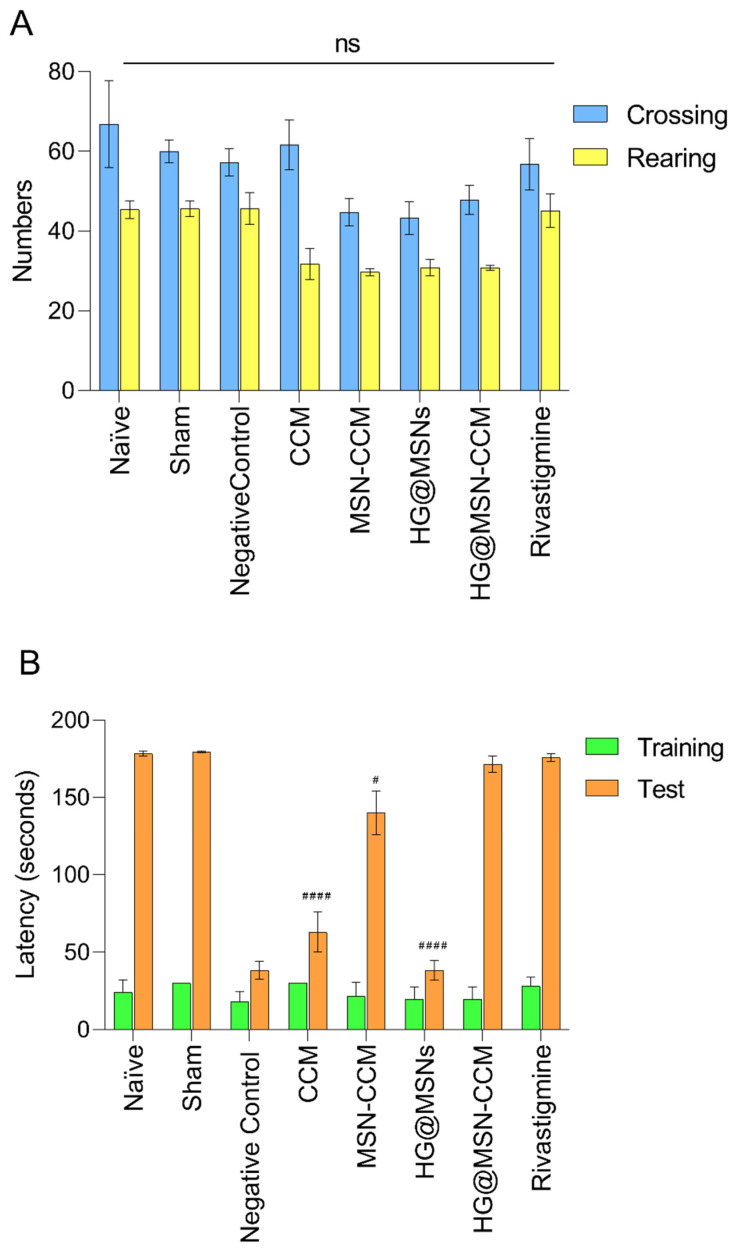
In vivo assay. Effects of treatment in the open field test (**A**) and inhibitory avoidance (**B**). Each bar represents the mean ± SD, from 6 to 12 animals per group. # *p* < 0.05, and #### *p* < 0.0001. The data were analyzed by the analysis of variance (ANOVA) and complemented by the Dunnett’s post hoc tests. ns = non-significant.

The results obtained in the open field test and inhibitory avoidance are shown in Figure 9A,B, respectively. In the open field test, behavioral parameters showing crossing and rearing were evaluated. The results indicated that the treatments did not cause changes in these parameters when compared to the control group (*p* > 0.05). That is, treatments did not cause sedative effects in the mice.

The results of the inhibitory avoidance demonstrated that, in the training session, no significant differences were observed between groups (*p* > 0.05). All mice spent about 12 to 30 s to get down on the platform and receive the shock. In the test session, groups that received i.c.v. injection of STZ (3 mg kg^−1^) and were treated with MSN-CCM, HG@MSN-CCM, and rivastigmine showed higher memory retention when compared with animals treated with CCM, HG@MSN (vehicle), being statistically significant (*p* < 0.05). These results show that nasal administration of MSN-CCM and HG@MSN-CCM reverse the cognitive deficits induced by i.c.v. injection of STZ.

## 4. Conclusions

In this study, mesoporous silica nanoparticles were obtained as nanocontainers for curcumin. MSNs with average size around 60 nm, high surface area, high pore volume, average pore diameter of 7.2 nm, and with high CCM encapsulation efficiency were developed. A temperature-responsive hydrogel was successfully developed displaying viscoelastic properties (allowing easy administration), mucoadhesive properties that overcome the mucociliary clearance mechanism, and good mechanical properties being considered adequate to enable intranasal administration. The nanosystem (MSNs and MSN-CCM) and the final formulation (HG@MSN-CCM) were considered biocompatible according to the in vitro results. The ex vivo permeation assays confirmed that the HG@MSN-CCM increases the CCM permeation compared with MSN-CCM through the porcine nasal mucosa. The in vivo assay showed that the MSN-CCM, as well as the HG@MSN-CCM, were able to revert the cognitive deficit in mice in a streptozotocin-induced Alzheimer’s disease (AD) model. These results confirm the potential of the formulations MSN-CCM and HG@MSN-CCM as a promising and innovative approach for AD treatment.

## Figures and Tables

**Table 1:** Surface area, average pore diameter, and pore volume from N_2_ adsorption–desorption isotherm measurements for MSNs and MSN-CCM.

Sample	Surface Area (m^2^ g^−1^)	Pore Diameter (nm)	Pore Volume (cm^3^ g^−1^)
MSNs	745	7.2	1.46
MSN-CCM	556	6.4	1.29

**Table 2:** Flow behavior (*n*) and consistency index (K) of the hydrogels.

Formulation	*n*	K (Pa s^−1^)	Hysteresis Area (Pa s^−1^)
HG	0.860	1.21	−239.652
HG@MSN-CCM	0.844	1.19	−209.964

**Table 3:** Mechanical and mucoadhesion properties of the HG and HG@MSN-CCM. The values are shown as mean ± standard deviation.

Formulation	Mechanical Parameters				Mucoadhesion	
	Hardness (mN)	Compressibility (mN s^−1^)	Adhesiveness (mN s^−1^)	Cohesiveness (Dimensionless)	S_MA_ (N)	w_MA_ (N s)
HG	11.97 ± 0.80	97.15 ± 6.95	91.08 ± 6.04	0.89 ± 0.02	0.183 ± 0.01	0.387 ± 0.02
HG@MSN-CCM	12.09 ± 0.62	98.15 ± 2.65	91.94 ± 3.63	0.90 ± 0.02	0.181 ± 0.01 *	0.383 ± 0.01 *

* *p* < 0.05 (unpaired *t*-test). Each parameter was analyzed separately (*n* = 5).

**Table 4:** Qualitative results of cytotoxicity study.

Samples	Halo (cm)	Cytotoxicity
Control (+)	1.267 ± 0.047	Severe
Control (−)	0.000 ± 0.000	Absent
CCM	0.000 ± 0.000	Absent
MSNs	0.000 ± 0.000	Absent
MSM-CCM	0.000 ± 0.000	Absent
HG	0.300 ± 0.000	Slight
HG-MSNs	0.267 ± 0.047	Slight
HG@MSN-CCM	0.233 ± 0.047	Slight

## Data Availability

Data are contained within the article and Supplementary Material.

## Supplementary Materials

The following supporting information can be downloaded at: www.mdpi.com/1999-4923/14/9/1976/s1, Figure S1. Graphics of the particle size distribution obtained by DLS from (A) MSNs and (B) MSN-CCM. Figure S2. Particle size distribution histograms (concentration versus size) obtained by NTA from (A) MSNs and (B) MSN-CCM. Figure S3. 3D graphs (intensity versus size) obtained by NTA from (A) MSNs and (B) MSN-CCM. Figure S4. Graphs of zeta potential distribution obtained by DLS from (A) MSNs and (B) MSN-CCM. Figure S5. FTIR spectra of CTAB, MSNs prior to CTAB removal and MSNs after to CTAB removal (green, red, and blue, respectively). Figure S6. FTIR spectra of (A) CCM and (B) MSNs and MSN-CCM (blue and red lines, respectively). Figure S7. Nitrogen adsorption–desorption isotherms of the (A) MSNs and (B) MSN-CCM.
