# Supplementary material for: Curcumin-Loaded Mesoporous Silica Nanoparticles Dispersed in Thermo-Responsive Hydrogel as Potential Alzheimer Disease Therapy
Source: Pharmaceutics. 2022 Sep 19;14(9):1976. doi: 10.3390/pharmaceutics14091976 (PMC9504573; doi:10.3390/pharmaceutics14091976)
Supplement: Supplementary file 1 [file pharmaceutics-14-01976-s001.zip › pharmaceutics-1892778-supplementary.pdf]

Supplementary information

Article

Curcumin-Loaded Mesoporous Silica Nanoparticles Dispersed in Thermo-Responsive Hydrogel as Potential Alzheimer Disease Therapy

Tais de Cassia Ribeiro [1], Rafael Miguel Sábio [1,*], Marcela Tavares Luiz [1], Lucas Canto de Souza [1], Bruno Fonseca-Santos [2], Luis Carlos Cides da Silva[3], Márcia Carvalho de Abreu Fantini[3], Cleopatra da Silva Planeta [1], and Marlus Chorilli [1,*]

1 Department of Drugs and Medicines, School of Pharmaceutical Sciences, São Paulo State University (UNESP), Araraquara 14800-903, Brazil
2 Department of Biotechnology, Health Sciences Institute, Federal University of Bahia (UFBA), Salvador 40110-902, Brazil
3 Institute of Physics, University of São Paulo (IF-USP), São Paulo 05508-090, Brazil

* Correspondence: rafael.m.sabio@unesp.br (R.M.S.); marlus.chorilli@unesp.br (M.C.); Tel.: +55-16-3301-6961

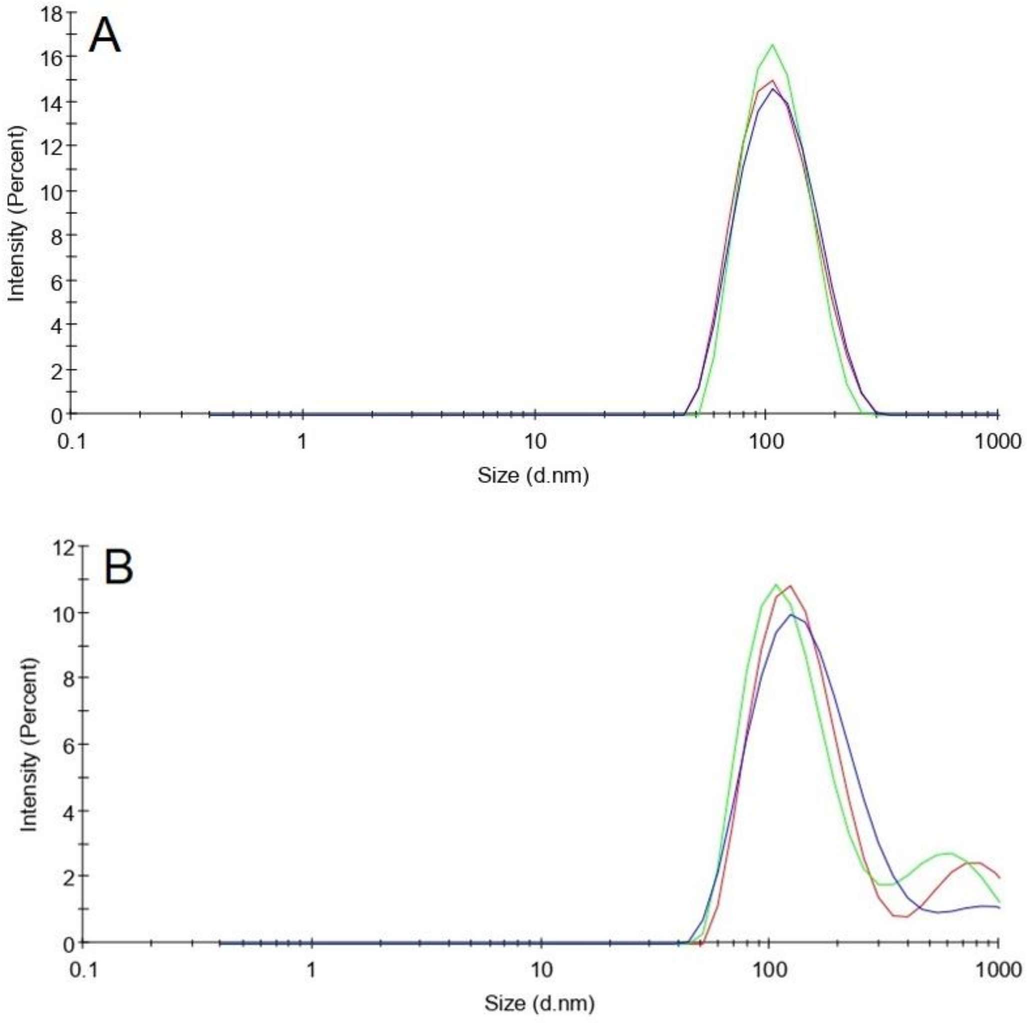

Figure S1. Graphics of the particle size distribution obtained by DLS from (A) MSNs and (B) MSN-CCM.

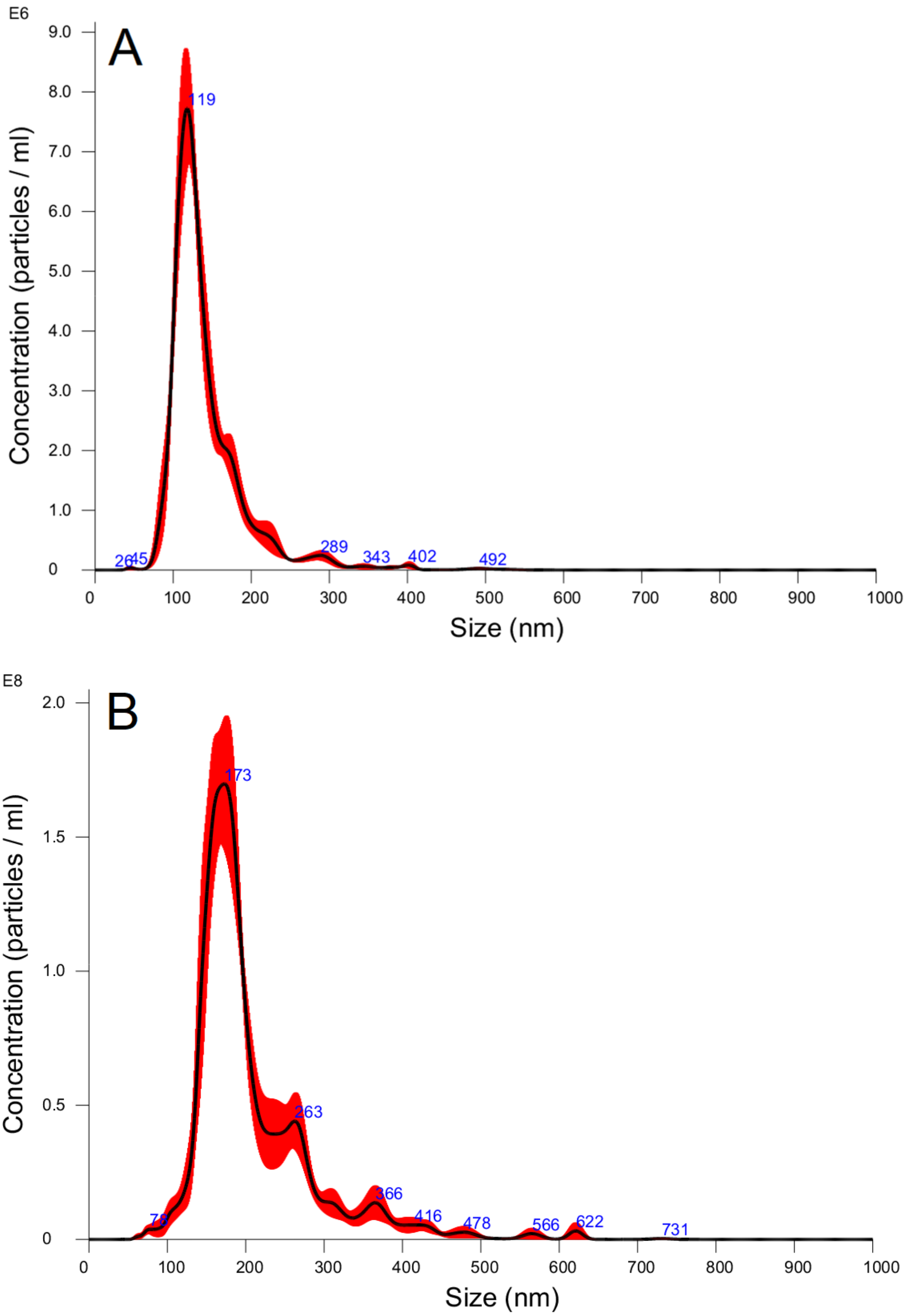

Figure S2. Particle size distribution histograms (concentration versus size) obtained by NTA from (A) MSNs and (B) MSN-CCM.

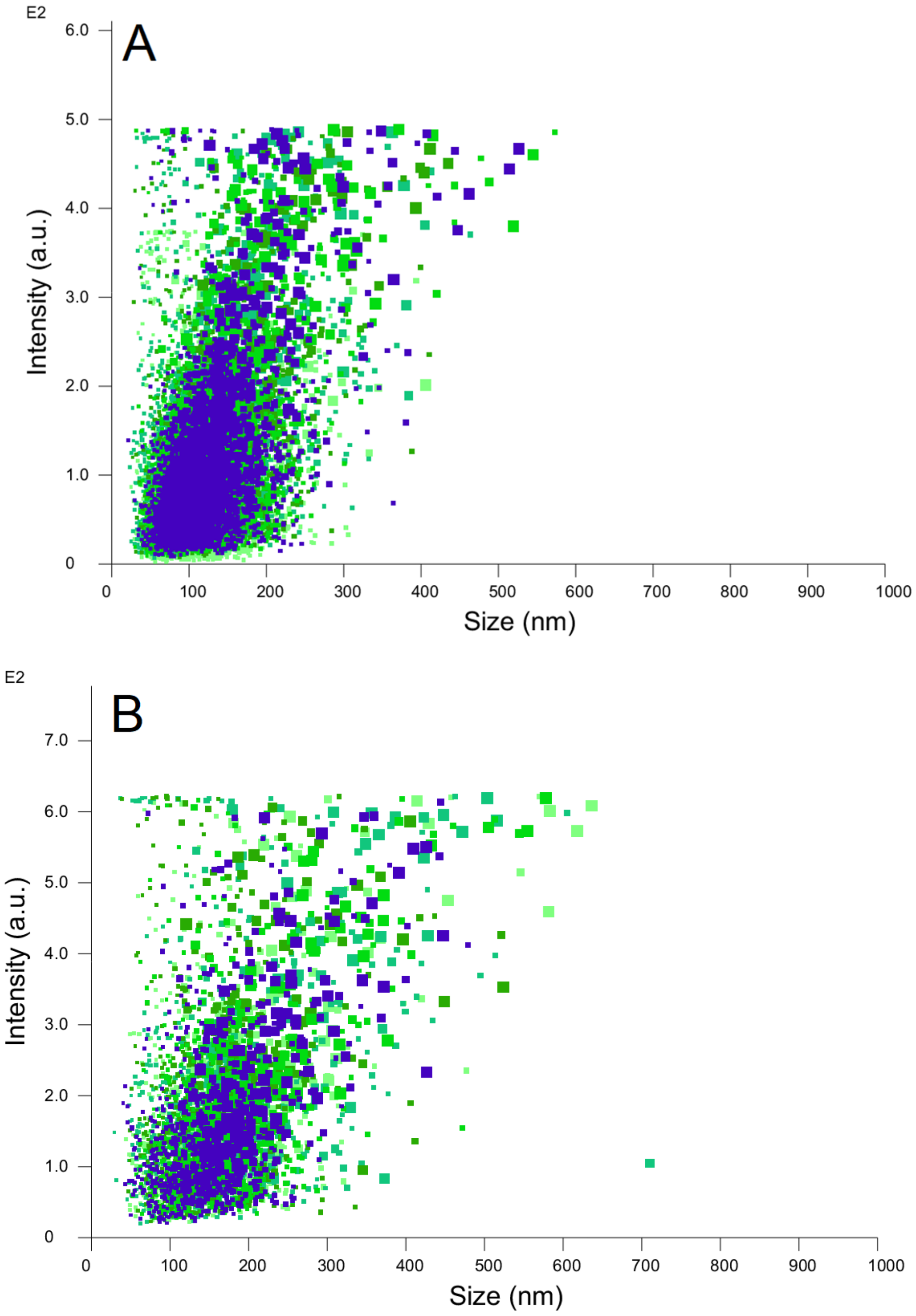

Figure S3. 3D graphs (intensity versus size) obtained by NTA from (A) MSNs and (B) MSN-CCM.

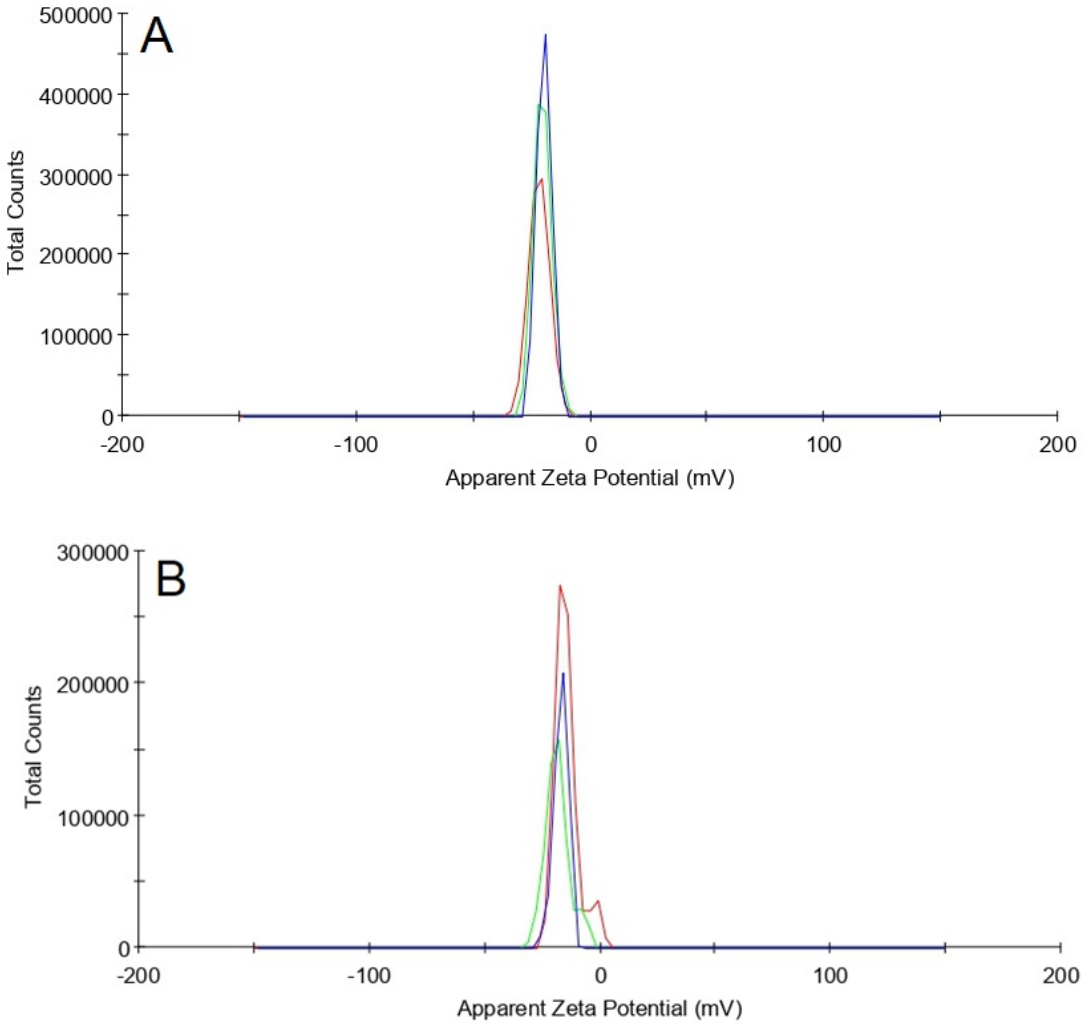

Figure S4. Graphs of zeta potential distribution obtained by DLS from (A) MSNs and (B) MSN-CCM.

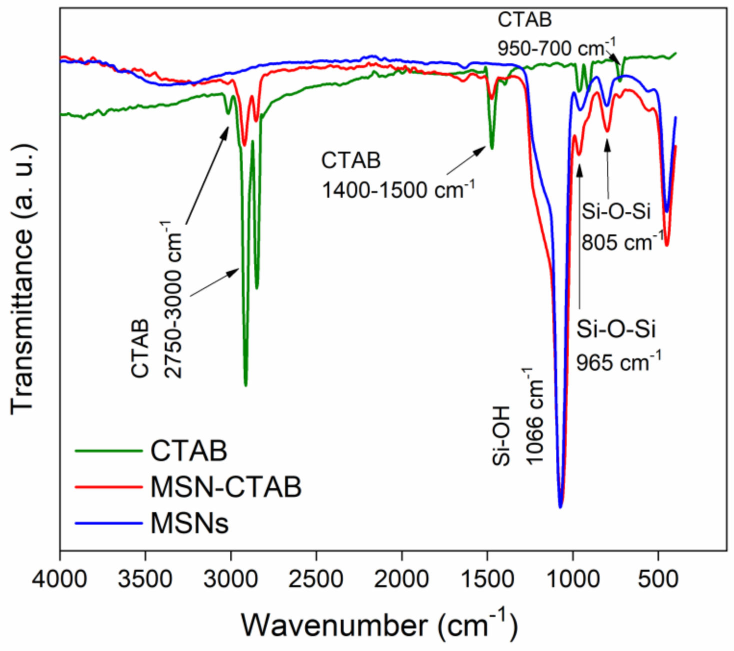

Figure S5. FTIR spectra of CTAB, MSNs prior to CTAB removal and MSNs after to CTAB removal (green, red and blue, respectively).

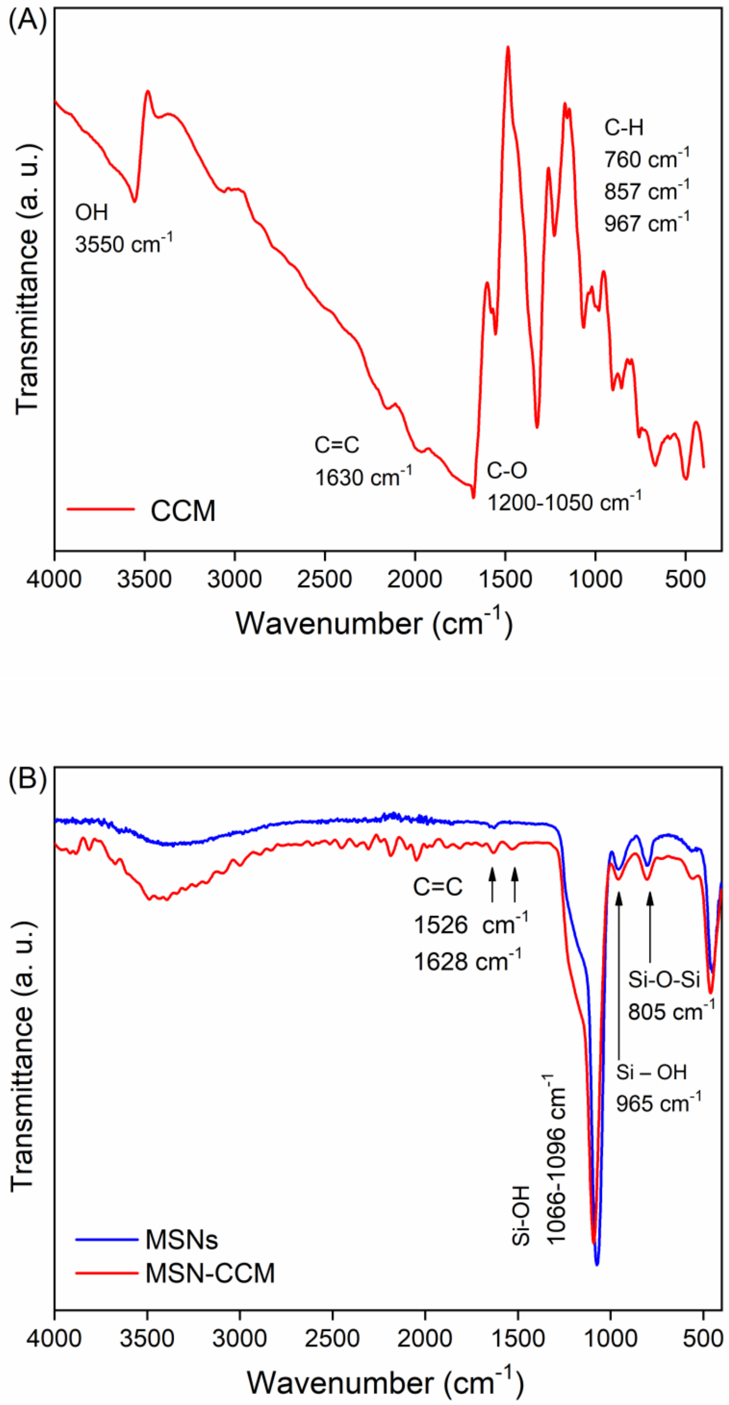

Figure S6. FTIR spectra of (A) CCM and (B) MSNs and MSN-CCM (blue and red lines, respectively).

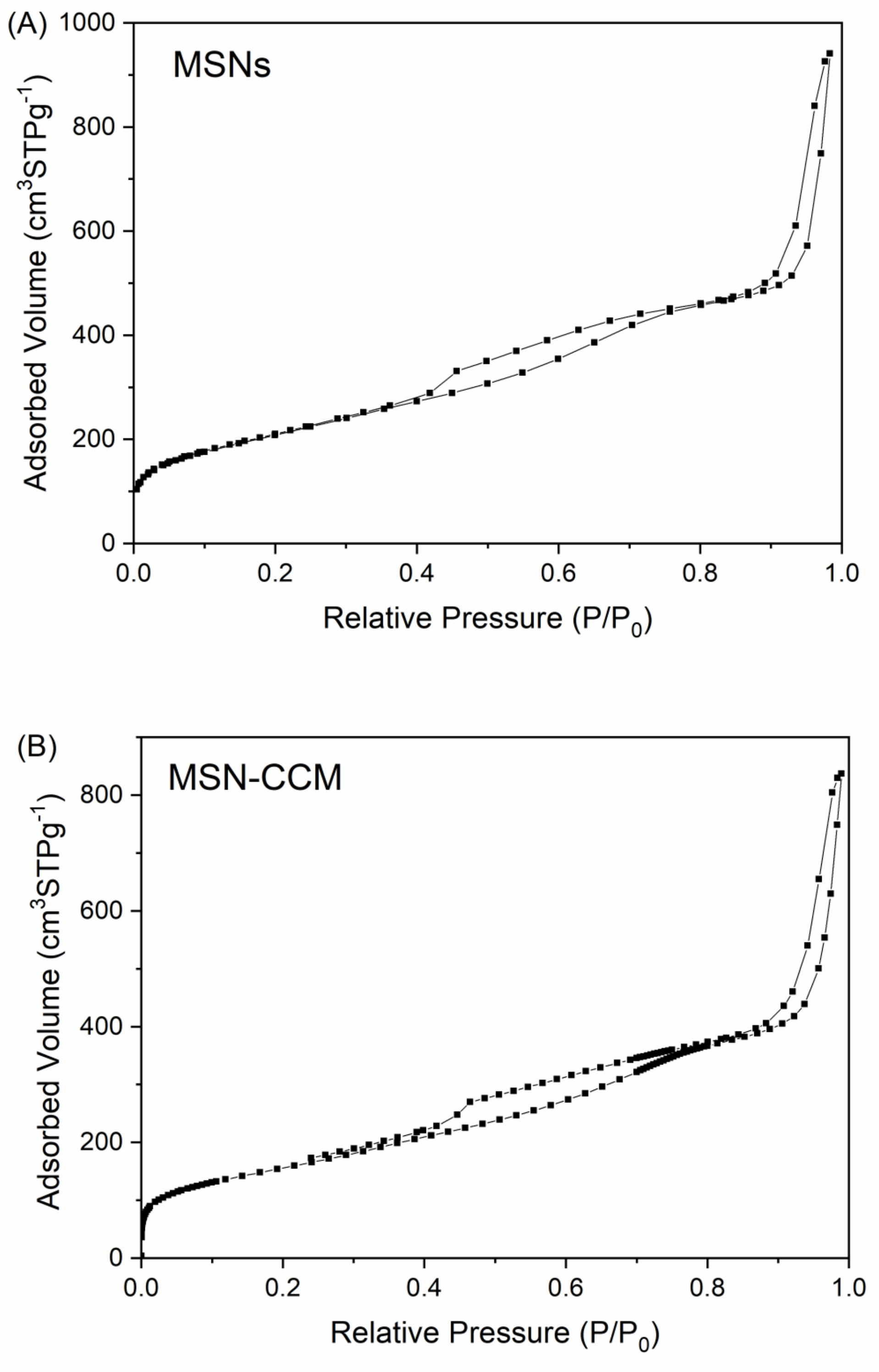

Figure S7. Nitrogen adsorption-desorption isotherms of the (A) MSNs and (B) MSN-CCM.
